# Immunotherapeutic Targeting of GPC3 in Pediatric Solid Embryonal Tumors

**DOI:** 10.3389/fonc.2019.00108

**Published:** 2019-02-26

**Authors:** Michael V. Ortiz, Stephen S. Roberts, Julia Glade Bender, Neerav Shukla, Leonard H. Wexler

**Affiliations:** Department of Pediatrics, Memorial Sloan Kettering Cancer Center, New York, NY, United States

**Keywords:** glypican 3, hepatoblastoma (HB), germ cell tumors (GCT), Wilms tumor (WT), rhabdoid tumor, rhabdomyosarcoma, neuroblastoma, immunotherapy

## Abstract

Glypican 3 (GPC3) is a heparan sulfate proteoglycan and cell surface oncofetal protein which is highly expressed on a variety of pediatric solid embryonal tumors including the majority of hepatoblastomas, Wilms tumors, rhabdoid tumors, certain germ cell tumor subtypes, and a minority of rhabdomyosarcomas. Via both its core protein and heparan sulfate side chains, GPC3 activates the canonical Wnt/β-catenin pathway, which is frequently overexpressed in these malignancies. Loss of function mutations in *GPC3* lead to Simpson-Golabi-Behmel Syndrome, an X-linked overgrowth condition with a predisposition to GPC3-expressing cancers including hepatoblastoma and Wilms tumor. There are several immunotherapeutic approaches to targeting GPC3, including vaccines, monoclonal antibodies, antibody-drug conjugates, bispecific antibodies, cytolytic T lymphocytes, and CAR T cells. These therapies offer a potentially novel means to target these pediatric solid embryonal tumors. A key pediatric-specific consideration of GPC3-targeted immunotherapeutics is that GPC3 can be physiologically expressed in normal tissues during the first year of life, particularly in the liver and kidney. In summary, this article reviews the current evidence for targeting childhood cancers with GPC3-directed immunotherapies.

## Introduction

Glypican 3 (GPC3) is an oncofetal protein which is enriched on the surface of several pediatric solid embryonal tumors. This mini review evaluates the biological role of GPC3, synthesizes the published expression data in pediatric solid embryonal tumors, and describes the current immunotherapeutic approaches to target GPC3.

## Biology

Glypicans are a highly conserved family of heparan sulfate proteolgycans which are attached to the plasma membrane via a C-terminal glycosyl-phosphatidylinositol (GPI) anchor (1, 2). These surface proteins interact with growth factors to influence morphogenesis and are predominantly expressed during development (1, 2). Six glypicans (numbered 1–6) have been identified in humans and broadly are subdivided into two groups with GPC1, GPC2, GPC4, and GPC6 are the orthologs of *Dally* whereas GPC3 and GPC5 are the orthologs of *Dlp* in *Drosophila melanogaster* (1).

*GPC3* is located on Chromosome Xq26 and encodes GPC3, also known as DGSX, GTR2-2, MXR7, OCI-5, SDYS, SGB, SGBS, and SGBS1 (2–4). During development, GPC3 is expressed in the placenta, fetal liver, fetal lung, and fetal kidney although it is absent or only minimally expressed in most adult tissues (5). This physiologic change may be mediated by suppression from DNA methylation within the *GPC3* promoter region (5–7).

GPC3 consists of an N-terminal domain that includes a secretory signal peptide as well as a GPI anchored C-terminal core protein containing two heparan sulfate chains (2–4). As with other glypicans, the GPC3 core protein and heparan sulfate side chains interact with a variety of regulatory proteins important in cell growth and differentiation, including Wnt, Hedgehog, and fibroblast growth factor (FGF) (8–12). In particular, GPC3 has been shown to interact with Wnts and binds directly to Frizzled, stimulating the formation of signaling complexes between these proteins which activates the canonical Wnt/β-catenin signaling pathway (8, 10). This signaling pathway is important for normal development of the kidney and liver, and is frequently aberrantly overexpressed in pediatric embryonal tumors (3, 8, 10, 13–17).

Simpson-Golabi-Behmel Syndrome (SGBS) is an X-linked overgrowth condition similar to the more common Beckwith-Wiedemann syndrome, and is associated with renal, hepatic, skeletal, and cardiac anomalies as well as predisposition to Wilms tumor, hepatoblastoma, and neuroblastoma (2, 18). SGBS is caused by constitutional microdeletions or truncating point mutations in *GPC3* which are predicted to result in a loss of function (2, 7, 18–21). Loss of GPC3 binding to insulin like growth factor 2 (IGF-2) was originally understood to cause this overgrowth phenotype but a series of subsequent papers demonstrates that this instead due, at least in part, to hyperactivation of Hedgehog signaling (20–24).

## Pediatric Tumors

Pediatric malignancies derived from tissues that express GPC3 during development, such as the liver or kidney, frequently demonstrate upregulation of GPC3 which is likely important to both malignant transformation and tumorigenesis in these childhood cancers. GPC3 drives cell growth and inhibits differentiation via alterations in Wnt/β-catenin, Hedgehog, and FGF signaling which are often aberrantly expressed in pediatric embryonal tumors. In addition, alternative pathways not involved in physiologic GPC3 function, such as the Yap-Hippo pathway as has been shown in adult liver tumors, may also contribute to GPC3-mediated pediatric tumor development (25, 26). Finally, GPC3 has been reported to increase expression of the multi-drug resistance associated protein and therefore GPC3 in tumors may contribute to chemoresistance and treatment failure (27–29).

**Figure 1:**
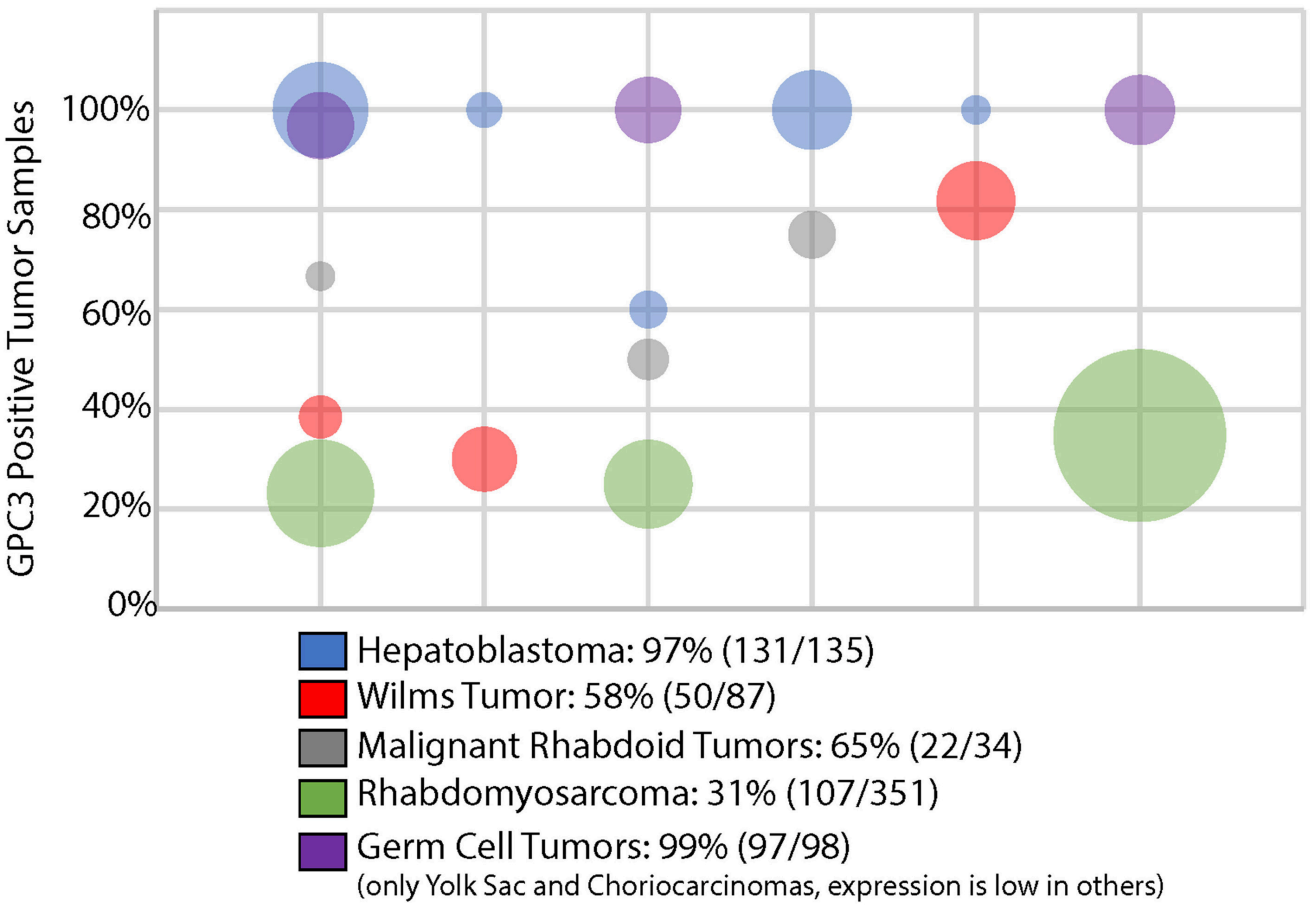
GPC3 immunohistochemistry in pediatric solid embryonal tumors. Bubble area is proportionate to the number of tumors evaluated in a particular study.

It is not fully understood how these childhood cancers are able to re-induce GPC3 expression. A study of the *GPC3* promoter methylation in primary pediatric embryonal tumors revealed gain of methylation mainly in boys with Wilms tumor and loss of methylation exclusively in girls with neuroblastoma (6). Increased tumor GPC3 expression was more commonly reported in a study of women than men with hepatocellular carcinoma (HCC), the most common adult liver tumor, although this has not been reproduced in subsequent studies (5). Thus, regulation of this X-linked gene may be not only age and tissue-specific but also gender-dependent and there are likely multiple means by which GPC3 becomes aberrantly deregulated in cancer. Nevertheless, across multiple studies, the extent of immunohistochemical (IHC) expression of GPC3 is relatively consistent for any given histology of embryonal tumor (Figure 1), each of which is to be reviewed in detail below.

### Hepatoblastoma

There are a variety of studies that demonstrate that GPC3 is nearly universally expressed on most hepatoblastomas although may be absent in less typical subtypes (e.g., teratoid) or portions of hepatoblastoma with mesenchymal differentiation (30–36). GPC3 was the second most highly transcriptionally overexpressed gene in a study of 48 hepatoblastoma tumors compared to normal liver (37). Although highly expressed, multiple studies have found that soluble GPC3 is an inferior serum biomarker of hepatoblastoma response compared with alpha fetoprotein, the current standard of care (37, 38). Combining the results from 5 studies evaluating GPC3 expression via IHC in hepatoblastoma found that 131/135 (97%) cases demonstrate GPC3 expression, as shown in Figure 1 (31–35).

### Germ Cell Tumors

Several studies of extragonadal germ cell tumors demonstrate that yolk sac tumors and choriocarcionomas virtually always express GPC3 via IHC (Figure 1) (35, 39–41). In fact, GPC3 expression has been used to distinguish ovarian germ cell tumors from ovarian carcinomas (39). Other germ cell tumors, such as teratomas, embryonal carcinomas, and germinomas rarely express GPC3 (40, 41).

### Wilms Tumors

Elevated transcriptional and proteomic expression of GPC3 is evident in a significant portion of Wilms tumors, as compared with adult kidney tumors and normal kidney tissue (36, 42, 43). Combining the results from 3 studies evaluating GPC3 expression in Wilms tumor revealed that 50/87 (58%) exhibit GPC3 expression, as shown in Figure 1 (34, 35, 43). In addition to constitutional mutations seen in patients with SGBS, somatic tumor mutations in GPC3 have even been identified in some cases of Wilms tumors (44).

### Rhabdoid Tumors

A series of 3 studies of extracranial malignant rhabdoid tumors demonstrate that 22/34 (65%) of these rare and highly aggressive tumors express GPC3 (34, 45, 46). Interestingly, other extrarenal INI1 negative solid tumors except for undifferentiated sarcomas rarely express GPC3 (45). Given the challenging diagnostic overlap between some of these INI1 negative tumors, particularly extrarenal malignant rhabdoid tumor and epithelioid sarcoma, GPC3 may not only be a reasonable therapeutic target but may be helpful in improving diagnostic accuracy (45).

### Rhabdomyosarcomas

A significant minority of rhabdomyosarcomas express GPC3, specifically 107/351 (31%) cases were positive in 3 different studies including both embryonal and alveolar subtypes (11, 34, 35, 47). Other pediatric sarcomas often do not express GPC3 (11, 47). Of all the glypicans, GPC3 exhibits the greatest homology with glypican 5 (GPC5), which is located on 13q32, a region of frequent genomic amplification in rhabdomyosarcomas and specifically associated with the PAX7-FOXO1 fusion (2, 11). Specifically in rhabdomyosarcomas, GPC5 has been specifically shown to potently activate Hedgehog signaling, which may be a result of its increased numbers of highly sulfated glycosaminoglan side chains compared with GPC3 (11, 48).

### Neuroblastomas

There is mixed evidence regarding the role of GPC3 in neuroblastoma, with some studies showing increased expression in patients with 4S disease but most revealing absent expression of GPC3 in nearly all cases (34, 35, 42, 49, 50). The related glypican 2 (GPC2), however, has been shown to be an oncoprotein and immunotherapeutic target in high risk neuroblastoma (51, 52).

## Treatments

The development of GPC3-directed targeted therapies was stimulated by research into HCC where GPC3 was not only present, but also noted to be a prognostic biomarker in adults (4, 5, 12, 53, 54). These therapies have included vaccines, monoclonal antibodies, antibody-drug conjugates, bispecific antibodies, cytolytic T lymphocytes (CTL), and chimeric antigen receptors, which are described in more detail below.

### Vaccines

From 2007 to 2009, a nonrandomized, open-label, phase I clinical trial with dose escalation of an HLA-A^*^24:02–restricted GPC3_298−306_ peptide vaccine enrolled 33 Japanese adults with advanced HCC (UMIN 000001395) (12, 55). The vaccine elicited a GPC3-specific CTL response in 30 patients, notably with 1 partial response and 19 with stable disease 2 months after initiation of treatment (12, 55). Following this an open label, single arm, phase II study was performed in advanced HCC patients in Japan using the HLA-A^*^24:02–restricted GPC3_298−306_ or HLA-A2-restricted GPC3_144−152_ peptide vaccine (UMIN 000002614) (12, 56). Although this study did not reach its primary endpoint, the 1 and 2 year event free survival was lower for the patients who underwent surgery alone as compared with those who received surgery plus vaccination (12, 56). This was statistically significant in a subgroup analysis of patients with GPC3 positive HCC (12, 56). These HLA-A^*^24:02 HLA-A2 GPC3-directed peptide vaccines were also used to treat Japanese patients with chemoresistant ovarian clear cell carcinoma (OCCC) (UMIN 000003696) (12, 57). This vaccine elicited a GPC3-specific CTL response in 15 of 24 patients who had peripheral blood mononuclear cells collected 3 times or more and 3 patients demonstrated a partial response (12, 57). Finally, a pediatric phase I study using these GPC3 directed vaccines was conducted in Japan and was found to be safe with a 2 month disease control rate of 66% (UMIN 000006357) (12, 58). A GPC3-specific CTL response was identified in 39% of patients in this study, the majority of whom were in remission and were diagnosed with hepatoblastoma (12, 58). To date, this is the only completed GPC3-directed immunotherapeutic clinical trial in pediatrics, as shown in Table 1.

**Table 1:** GPC3-targeted cancer immunotherapy trials.

**Therapy name (Drug type)**	**Phase**	**Trial number**	**Eligibility**	**Status**	**Sponsor**	**Country**
GPC3 Peptide Vaccine	I	UMIN 000001395	Adult HCC	Complete	National Cancer Center Hospital East	Japan
	II	UMIN 000002614	Adult HCC	Complete	National Cancer Center Hospital East	Japan
	II	UMIN 000003696	Adult OCCC	Complete	National Cancer Center Hospital East	Japan
	I	UMIN 000006357	**Pediatric** GPC3+ Tumors	Complete	National Cancer Center Hospital East	Japan
Codrituzumab (Monoclonal Antibody)	I	NCT 00746317	Adult HCC	Complete	Chugai Pharmaceutical	USA
	I	JapicCTI 101255	Adult HCC	Complete	Chugai Pharmaceutical	Japan
	I^*^	NCT 00976170	Adult HCC	Complete	Chugai Pharmaceutical	USA
	II	NCT 01507168	Adult HCC	Complete	Hoffman-La Roche	USA
	I^**^	JapicCTI 163325	Adult HCC	Open	Chugai Pharmaceutical	Japan
ERY974 (Bispecific Antibody)	I	NCT 02748837	Adult HCC	Open	Chugai Pharmaceutical	Multi-National
GAP T cells (CAR T Cell)	I	NCT 02932956	**Pediatric** GPC3+ Liver Tumors	Open	Baylor College of Medicine	USA
GLYCAR T cells (CAR T Cell)	I	NCT 02905188	Adult HCC	Open	Baylor College of Medicine	USA

* *Combination with Sorafenib*.

** *Combination with Atezolizumab*.

*Bold text refers to pediatric studies*.

### Monoclonal Antibodies

Codrituzumab (RO5137382; RG7686; GC33) is a recombinant humanized antibody targeting GPC3 which interacts with CD16/FcγRIIIa on natural killer (NK) cells to cause antibody-dependent cytotoxicity in a GPC3-dependent manner (59–63). This drug has been studied in a series of 4 clinical trials in adults with HCC. For the first-in-man study in the US, 20 patients with advanced HCC were enrolled on a dose-escalation study of codrituzumab and a maximum tolerated dose was not reached as there were no dose limiting toxicities up to the highest planned dose level of 20 mg/kg weekly (61). Time to progression was statistically significantly higher in those HCC patients with higher GPC3 expression (61). A subsequent Japanese phase I study in advanced HCC patients revealed that 7/13 (54%) patients had stable disease, 3 of whom had prolonged (>5 month) disease stabilization (JapicCTI-101255) (62). In a phase Ib study in combination with sorafenib (NCT00976170), codrituzumab was not found to provide clinical benefit although this study demonstrated that ^124^I radiolabeled codrituzumab was useful to monitor antibody uptake in the tumor and persistence of GPC3 expression after treatment (60). In a randomized placebo controlled phase II study (NCT01507168), codrituzumab similarly did not show a clinical benefit in advanced HCC patients, however combined elevation of tumor GPC3 and CD16/FcγRIIIa on NK cells correlated with survival (63–65). In HCC, expression of GPC3 has been shown to be a poor prognostic feature (66). Thus, even in these highest risk HCC patients with GPC3 expression, codrituzumab may provide clinical benefit, although monotherapy alone appears to be inadequate for HCC. Given the effectiveness of checkpoint inhibition with HCC, a combination of codrituzumab with the PD-L1 inhibitor atezolizumab is currently being evaluated in a Japanese phase I study of adult HCC patients (JapicCTI-163325). To date, codrituzumab is the only GPC3-directed immunotherapy to have a completed a clinical trial in the United States, as shown in Table 1.

The Ho Lab at the National Cancer Institute (Washington, DC, USA) has generated several additional GPC3-directed antibodies which have been extensively studied preclinically, including HN3, YP7, and HS20. HN3 is a GPC3-directed antibody that recognizes a cryptic Wnt binding domain and causes cell cycle arrest in HCC models via inactivation of Yap signaling (67). YP7 is another high affinity monoclonal antibody directed to the cell surface bound GPC3 and exhibited significant growth inhibition in HCC xenografts (68). HS20 is a human monoclonal antibody that recognizes the interaction site between the C-terminal GPC3 core fragment and heparan sulfate side chains in order to disrupt their interactions with Wnt (13, 69). This antibody was found to be an effective inhibitor of Wnt/β-catenin signaling *in vitro*, effectively inhibited HCC xenograft growth *in vivo*, and further was shown to impair cell migration and motility (13, 70).

### Antibody-Drug Conjugates

Since GPC3 is efficiently internalized, it also is a good candidate for conjugation of antibodies to toxins (71). As a result, HN3 and YP7 were conjugated to the Pseudomonas endotoxin A and shown to be effective at reducing growth of xenografts *in vivo*, although notably the HN3-based drug conjugate, which is able to interfere with Wnt/β-catenin signaling, was more effective preclinically (71). There was significant *in vivo* toxicity so key immunogenic epitopes were removed from this antibody-drug conjugate, termed HN3-mPE24, in order to make it clinically viable (72).

### Bispecific Antibodies

ERY974 is a bispecific antibody which targets both GPC3 (it was notably generated from codrituzumab) and CD3 and demonstrates *in vivo* antitumor efficacy against several GPC3 positive tumors (73). Intriguingly, ERY974 was effective even against tumors with nonimmunogenic features, by causing inflammation in the local tumor microenvironment (73). This is an important observation as even tumors which are not traditionally understood to be immunologically targetable on the basis of increased neoantigen expression could potentially be treated using this approach. More recently, Sano and colleagues presented results of a follow-up study which demonstrated synergy between ERY974 with Paclitaxel and Cisplatin (74). Given that Cisplatin is already an effective treatment modality for the majority of the GPC3 expressing pediatric solid embryonal tumors, this represents a promising opportunity for future combination studies. As shown in Table 1, an adult multicenter international phase I clinical trial of ERY974 is currently open in the United States and Europe (NCT02748837) and has planned expansion cohorts for stomach, esophageal, and other GPC3-expressing cancers.

### Cytolytic T Lymphocytes

During the aforementioned peptide vaccination clinical trials, as well as a clinical study of HCC patients (UMIN 000005093), multiple peptide specific CTL clones were generated from peripheral blood and tumor tissue (12). These third party T cells are actively being developed for adoptive immune cell treatment of GPC3-positive tumors, as has been effectively utilized in the treatment of EBV associated post-transplantation lymphomas (12, 75, 76).

### Chimeric Antigen Receptors

The Heczey Lab at Baylor College of Medicine (Houston, TX, United States) has generated several GPC3-targeted chimeric antigen receptor (CAR) constructs (77). Notably all of these GPC3/CARs rendered T cells highly cytotoxic to GPC3-positive HCC, hepatoblastoma, and malignant rhabdoid tumor cell lines *in vitro* as well as HCC and malignant rhabdoid tumors *in vivo* (77). The GPC3 directed CAR with the 4-1BB Zeta chain was the most effective at inducing T cell expansion and proliferation (77). As a result, two clinical trials are currently in development, GLYCAR T cells (NCT02905188) for adults with HCC and GAP T cells (NCT02932956) for children aged 1–21 with GPC3 positive liver tumors (Table 1).

## Challenges

Although GPC3 is expressed in a wide variety of pediatric solid tumors, it is also expressed physiologically in infants, predominantly in the liver and kidney, with detectable serum levels during the first year of life (35). Thus, GPC3 targeted therapies could cause significant toxicity not seen in adults thus far due to persistent physiologic expression of GPC3 in the liver and kidney. If indeed clinical trials in pediatrics reveal immunogenic targeting of normal tissues, strategies to limit toxicity will need to be employed, such as limiting age to children >1 year of age as is being done in the GAP T cell study (NCT02932956). Given the generalized expression of GPC3 in the fetus and placenta, GPC3 based immunotherapies are likely to be teratogenic. Care must be made when counseling and treating women of childbearing age with GPC3-based immunotherapies. Finally, immunotherapies targeting these cancers need to be designed such that they preferntially target the core C-terminal GPC3 protein, its heparan sulfate side chains, or their interactome rather than the soluble N-terminal GPC3. In fact, soluble GPC3 expression may be useful as a biomarker of response to GPC3 therapies.

## Conclusions

The heparan sulfate proteoglycan GPC3 is an attractive target for drug development as it is highly upregulated in HCC and several pediatric solid embryonal tumors and is responsible for driving key growth and developmental pathways which are currently not effectively targeted using our existing therapies (2, 12, 78). At this point, there is very limited clinical experience with GPC3-directed immunotherapeutics in pediatric oncology: A GPC3-directed vaccine study was conducted in Japan for children with solid tumors expressing GPC3 (UMIN 000006357) and in December 2018, the GAP CAR T cell study (NCT02932956) opened for children and young adults with GPC3-expressing liver tumors (12, 58). Vaccines, monoclonal antibodies, antibody-drug conjugates, bispecific antibodies, CTLs, and CAR T cell based therapies are all emerging treatment options which may provide enhanced ability to target GPC3 in pediatric solid embryonal tumors. As ongoing clinical trials in adults demonstrate which of these GPC3-based modalities are safe and beneficial, it is imperative that we rigorously evaluate the role of these potentially life-saving therapies in children and adolescents with GPC3-driven tumors.

## Author Contributions

MO generated the initial draft of the manuscript. All authors reviewed the manuscript for content and accuracy and are responsible for its content.

### Conflict of Interest Statement

The authors declare that the research was conducted in the absence of any commercial or financial relationships that could be construed as a potential conflict of interest.

## Abbreviations

CTL: Cytolytic T Lymphocytes
FGF: Fibroblast growth factor
GPC: Glypican
GPI: Glycosyl-phosphatidylinositol
HCC: Hepatocellular carcinoma
IGF: Insulin-like growth factor
IHC: Immunohistochemistry
NK: Natural killer
OCCC: Ovarian clear cell carcinomas
SGBS: Simpson-Golabi-Behmel Syndrome.
